# DKey software for editing and browsing dichotomous keys

**DOI:** 10.3897/zookeys.735.21412

**Published:** 2018-02-06

**Authors:** Adam Tofilski

**Affiliations:** 1 Department of Pomology and Apiculture, University of Agriculture in Krakow, Al. 29 Listopada 54, 31-425 Krakow, Poland

**Keywords:** key, single-access key, software, taxonomic identification of species

## Abstract

Despite advances in computer technology and the increasing availability of multiple-access taxonomic keys, traditional dichotomous keys remain the most often used taxonomic identification tools. On the other hand, there seems to be a lack of an editor of dichotomous keys, which is both freely available and easy to use. The DKey software was developed in order to alleviate this problem. A taxonomic key in text format can be imported to the software in order to edit it. Various editing options are possible, including: moving couplets, removing couplets, combining keys and renumbering keys. The software can output the key either in the traditional text format, ready for publication in a scientific journal, or in hypertext linked format, which makes identification faster and easier, due to the fact that pointers can be clicked in order to move to the next couplet. The DKey software should be useful for both taxonomic experts creating keys and those who use them for identification. The DKey software is freely available and open source.

## Introduction

The identification of species is often based on dichotomous keys (also called single-access keys or pathway keys) (Quicke 1993, Walter and Winterton 2007). They are a series of consecutively numbered couplets. Each couplet consists of two parts called leads. At the end of each lead, there is a reference, which can be either a number pointing to another couplet or a text indicating the name of a species or another taxonomic group. The two types of references are called “pointer” and “endpoint”, respectively. The identification based on a dichotomous key starts at couplet number one and it is stepwise. At each step, the user reads both leads of a couplet and chooses one that better fits the identified individual. If the chosen lead is associated with a pointer, then the user goes to another couplet, which is the next step of the identification. The identification is finished when the chosen couplet is associated with an endpoint.

Originally, dichotomous keys were constructed by taxonomic experts without any support form computer technology. However, when computers became more widespread, computer programs for the automated construction of taxonomic keys were developed (Pankhurst 1971, Dallwitz 1974, Quicke 1993). Computerized key construction software requires a data matrix consisting of a table with information about multiple characters of each taxon in the key. The key is constructed automatically by a computer algorithm; however, there is often the need for some intervention from the user to improve it. The data matrix can also be used for the construction of a free-access key (also called a multi-access key) (for review see Walter and Winterton 2007, Hagedorn et al. 2010, Cerretti et al. 2012). In this type of taxonomic key, there is no fixed sequence of identification steps. Instead, the user chooses characters from the data matrix that are easily available. This attitude can be preferred when some characters are not available in the identified specimen.

Multiple-access keys have many advantages (Dallwitz et al. 2000). In those keys not only qualitative but also quantitative characters can be used. Moreover, tolerance to errors is higher than in dichotomous keys. Despite their advantages multiple-access keys have not become overwhelmingly popular among taxonomist and manmade dichotomous keys remain the most often used identification tool (Quicke 1993, Walter and Winterton 2007). The preparation of the data matrix and, in particular, the fine tuning of the process of key construction requires some specialist knowledge. On the other hand, most taxonomists have limited interest in computer technology and sophisticated software can intimidate them. In consequence, many taxonomists design dichotomous keys without the use of any dedicated computer software, except a text editor. Creating a key in this way is time-consuming. A relatively simple task of numbering couplets is tedious and error prone. This task can be repeated many times when preparing the key, as any removal or addition of a couplet requires renumbering of some couplets and pointers. This problem is particularly acute in the case of large keys consisting of hundreds of couplets. There seems to be a lack of an editor of dichotomous keys, which is freely available and easy to use.

The aim of the project was to develop the DKey software, which would assist taxonomists in the preparation of dichotomous identification keys. A taxonomic key in text format can be easily imported to the software in order to edit it. The software can output the key either in traditional text format, ready for publication in a scientific journal, or hypertext linked format, which makes identification faster and easier. The DKey software is freely available and open source.

## Methods

DKey was developed in the C++ programming language. It is based on the QT framework. At the moment, executables are available for the Windows operating system (http://drawwing.org/dkey). However, users of macOS and Linux can obtain the source code and build executables for their operating system, as QT is a cross-platform framework. In future access to DKey should be equally easy for all three operating systems. The source code of the software can be downloaded from the GitHub (https://github.com/DrawWing/DKey). DKey is open source and it is licensed under GNU General Public License, version 3.

DKey allows for reticulation, which means that a couplet can be reached over more than one path and a single taxon can be associated with more than one endpoint. The only restrictions are that the couplet numbers need to be unique, they need to start with 1, they need to be consecutive, each couplet need to be reached from couplet 1 and it needs to refer either to an existent other couplet or an endpoint. The software does not create the key automatically; it is a responsibility of the taxonomic expert to create couplets and to arrange them in an optimal way. In order to test the software, I have used the existing key to the genera of Agathidinae (Hymenoptera, Braconidae) (Sharkey et al. 2009).

## Results

### Description of the software

**Figure 1.**
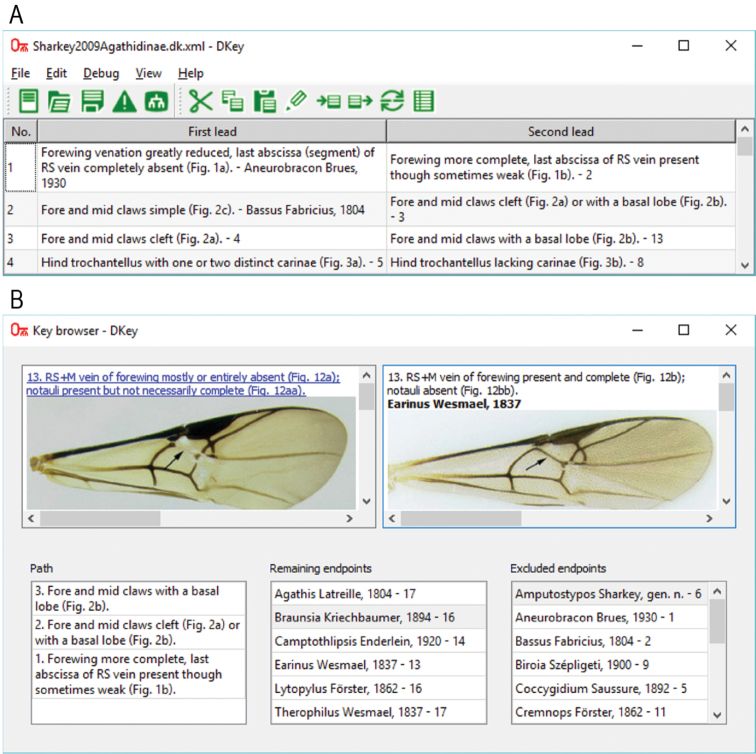
An example of using the DKey software based on the key to the genera of Agathidinae (Sharkey et al. 2009). The key was imported to the DKey software (**A**) and displayed in an interactive key browser (**B**).

The DKey software has a graphical user interface. In the main window, the taxonomic key is displayed in a table (Fig. 1A) where one row corresponds to one couplet. There are three columns. In the first column, there is the couplet number; in the second column, the first lead; and finally, in the third column, the second lead. The table can be used to edit the key. It is possible to insert, remove, copy, cut and paste couplets. Those editing operations can be made only within one key. If two keys need to be combined, the “append” option should be used. Then, the content of the appended file is added at the end of the currently edited key.

When couplets are inserted, removed, moved or copied, the key is not renumbered automatically and its consecutive numbering can be temporally broken. The user decides when the renumbering occurs. This makes it easier to keep track of changes made. Moreover, the renumbering can take a noticeable time in the case of large keys. The renumbering should be done before the key is exported or displayed in the key browser.

There is a validation tool allowing to find logical errors in the key. Among others it is verified if the couplet numbering is unique and consecutive, if the pointers are valid and if each couplet has at least one reference in other couplets. Moreover, a warning is generated whenever reticulation occurs in the key. The reticulation means that the same endpoint occurs in the key more than once or a single couplet is referred by more than one couplet. A key created with DKey can be saved in a XML file which allows relatively easy import to other software. Moreover, in this format international characters can be safely exchanged between operating systems.

DKey can format the key in various ways. For publication in a scientific journal, the key can be saved in rich text format. On the other hand, for making the key available online, it should be saved in hypertext linked format. In this format, pointers can be clicked in order to move to the next couplet. Moreover, a key browser (Fig. 1B) can be used on a local computer to make identification more user-friendly. In the key browser, only one couplet is displayed at any time. The key browser consists of five windows. The two top windows contain the two leads of the couplet and images associated with the leads. If a lead is associated with a pointer, there is a link to the pointed couplet. The three bottom windows of the key browser contain: the path, the remaining endpoints and the excluded endpoints. The path, or history of identification, contains a list of couplets leading to the current couplet. The remaining endpoints contain a list of taxa, which can be reached from the current couplet. The excluded endpoints contain list of taxa, which cannot be reached from the current couplet. At the beginning of identification, when the first couplet is displayed, the path window and the excluded endpoint window are empty; on the other hand, the remaining endpoint window contains all the taxa covered by the key. By following the steps of the identification and visiting couplets, the lists in the path window and the excluded endpoint window are growing and the list in the remaining endpoint window is shrinking until there are only two endpoints.

Identification using a key browser is easier because the user moves from couplet to couplet by clicking a chosen lead. It is also possible to go back to the earlier stages of identification by clicking on the list of steps leading to current couplet. Moreover, the software searches the leads for the keyword “Fig.”, and if found, it looks for the presence of an image file in the same directory. If the image is found, it is displayed in the key browser next to the lead in which it is referenced. This simple mechanism is sufficient for the integration of images into the key; there is no need for a manual linking of images with couplets.

### Getting started

The preparation of a new key can be started from scratch using the “new key” option. Then, the first dummy couplet, which needs to be edited, is created. In order to edit the couplet it should be double clicked. More couplets can be added using the option “insert couplet below” (for details see Suppl. material 1).

Taxonomic keys are often developed by the modification of older keys created by another expert. Therefore, the import of an existing key in text format is an important part of the software. Traditional dichotomous taxonomic keys are usually formatted in a consistent way (Quicke 1993); therefore, they can be analyzed by computer software in order to extract various information. In the language of computer technology, the analysis is called “parsing”. The DKey software parses the key in text format in order to extract all relevant information, including: number of the couplet, leads, pointers and endpoints. In order to facilitate the import, the user should place leads in separate lines and place a tab character in each line before pointers or endpoints. The process of import is not interactive and incorrect preparation of the imported file will result in incorrect key structure. However, the described earlier validation can be used to detect those errors. If the errors are present the user should edit the imported file and repeat the import.

In order to illustrate the import, the key to the genera of Agathidinae (Sharkey et al. 2009) was saved as a plain text file and small adjustments facilitating the import to the DKey software were made (for details see Suppl. material 2). The modifications included adding a tab character before each reference (both endpoint and pointer), adding the keyword “Fig.” before each figure number and removing unwanted line breaks within each lead. The modified text file was imported to the DKey software. In the imported key some errors were detected because there was one couplet (number two) with three leads. It was converted to two couplets with two leads in order to be compatible with the dichotomous key. Finally the key was renumbered in order to correct it for the added couplet in which the number was not consecutive (Fig. 1A, B).

## Discussion

The DKey software fills the gap for an easy-to-use and free editor of conventional dichotomous taxonomic keys. The main advantage of the DKey software is its wide availability. It can be downloaded free of charge and used by both scientists as well as a wider audience. The price of commercial software, for example Lucid Phoenix (Anonymous 2017), can be prohibitive for some enthusiasts of taxonomy. Not only is the DKey software free of change, but its source code is also widely available. Therefore, it can be developed in the future by a larger group of programmers who can correct and improve it. In consequence, the software can survive longer. Many projects related to biodiversity informatics vanished or stopped to be developed when the funding has finished or when the original developer lost interest in the further improvement of the software. In the case of open source software, there is a chance that programmers, other than the author, will continue the work on it.

There were other attempts to develop free and open source software for taxonomic keys. Open Key Editor (Martellos et al. 2010, van Spronsen et al. 2010) can be used to create user-friendly taxonomic keys available online or on mobile devices. This software is open source; however, the license under which it is distributed is relatively restrictive (Hagedorn et al. 2011). The installation of Open Key Editor can be difficult for most taxonomists, as it requires a web server, the creation of an SQL database and a manual modification of the configuration files.

There is also a wide range of freely available software, which can be used for the creation of online identification keys, including: ActKey (Brach and Song 2005), ARPHA Writing Tool (Smith et al. 2013), KeyBase (Thiele and Klazenga 2016), WEBiKEY (Attigala et al. 2016). The purpose of this software is to make identification easier. Taxonomic keys published in scientific journals and books are formatted for efficient printing and page layout. This formatting can create problems for the key users, as images are usually on a different page then the couplets referring to them. In order to minimize those problems, dichotomous keys can be implemented as a webpage or a computer program. In this form, the key is more user-friendly and the identification time can be markedly reduced because the user does not need to flip pages to find the next couplet or an image (Wright et al. 1995, Farr 2006, Burkmar 2014). However, the process of preparing the computer-based keys can be time-consuming because it requires the preparation of a data matrix or rewriting the key. On the other hand, DKey allows a relatively easy import of existing keys and images. A user of a traditional printed key can scan it, convert it to text and import it to the DKey software in order to make identification faster and more flexible. Moreover, taxonomic experts who use the software to create a key for scientific publication do not need to invest any more time or effort to create a computer-based version of the key.

In contrast to other free software, DKey is focused not only on the creation of computer based keys but also provides many editing options, which are not available elsewhere, including: import from text, export to text, combining keys, moving and copying couplets. The DKey software is particularly useful in the case of large taxonomic keys consisting of hundreds or thousands of couplets. Nowadays, the identification of large taxonomic groups involves using many small keys. There are separate keys for families, genera, species groups and species. The smaller keys are easier to manage by the taxonomic experts who created them because their renumbering is less time-consuming. On the other hand, this solution is less user-friendly because the user needs to find the key to the next taxonomic level. DKey can be easily used to combine many smaller keys into one very large one.

Taxonomists are encouraged to publish their datasets and allow others to re-use them in the future (Penev et al. 2008, 2009). The current recommendations (Penev et al. 2009) are related mainly to interactive keys based on data matrices and there is no standard format for dichotomous keys. The later keys are usually presented in two main formats: bracketed and indented (Quicke 1993). Within each of those formats, there are many variants with various punctuation marks around couplet numbers. This variation hinders the process of parsing a key in text format by a computer software. The use of standard formatting would make the exchange of information easier. The format in which the key is saved by the DKey software can become such a standard.

## Conclusion

The DKey software is an easy-to-use and freely available dichotomous taxonomic key editor. It can be used for importing existing keys, editing them and exporting them in various formats. Some of the formats are suitable for publication in scientific journals; others make identification easier and faster. The DKey software should be useful for both taxonomic experts creating keys and those who use them for identification.

## Supplementary materials

Supplementary material 1 Video tutorial describing how to build a simple taxonomic key using DKey software Data type: Multimedia. Explanation note: Use any video player which supports MP4 format in order to see the tutorial. This is an open access article distributed under the terms of the Creative Commons Attribution License (CC BY 4.0), which permits unrestricted use, distribution, and reproduction in any medium, provided the original author and source are credited.Author: Adam Tofilski

Supplementary material 2 Video tutorial describing how to import a taxonomic key to DKey software Data type: Multimedia. Explanation note: Use any video player which supports MP4 format in order to see the tutorial. This is an open access article distributed under the terms of the Creative Commons Attribution License (CC BY 4.0), which permits unrestricted use, distribution, and reproduction in any medium, provided the original author and source are credited.Author: Adam Tofilski
